# Hepcidin Is a Valuable Therapeutic Target for Colorectal Cancer

**DOI:** 10.3390/cancers16234068

**Published:** 2024-12-05

**Authors:** Rachele Frascatani, Marco Colella, Giovanni Monteleone

**Affiliations:** 1 Department of Systems Medicine, University of Rome “Tor Vergata”, 00133 Rome, Italy; rakfrasc@gmail.com (R.F.); marco.colella.bio@gmail.com (M.C.); 2 Gastroenterology Unit, Fondazione Policlinico “Tor Vergata”, 00133 Rome, Italy

**Keywords:** immunotherapy, immunogenic cell death, gasdermin, tumor microenvironment

## Abstract

Colorectal cancer (CRC) is one of the most frequent neoplasms and a major cause of cancer death worldwide. Despite recent advances in treatment approaches, the prognosis of advanced CRC remains poor, thus indicating the necessity of more effective treatments for CRC patients. CRC cells produce high levels of hepcidin, a peptide hormone that binds to the membrane-bound ferroportin and promotes its internalization and degradation, thus sequestering iron into the cancer cells with the downstream effect of enhancing tumor growth. Additionally, CRC cell-expressed hepcidin prolongs cell survival and, by targeting both CD8+ T cells and myeloid cells, restrains the induction of an efficient immune response against tumor antigens. The greatest expression of hepcidin is found in patients with metastatic CRC, and CRC patients with high hepcidin content have a worse survival rate than those with low hepcidin content. In the present article, we review the data supporting the prominent role of hepcidin in colon tumorigenesis and discuss how hepcidin inhibitors can help treat CRC patients in the metastatic setting with particular regard to the impact of hepcidin modulation on immunotherapeutic outcomes.

## Simple Summary

Hepcidin is a peptide hormone classically known as a regulator of iron balance. Hepcidin is mainly produced in the liver, but recent studies have shown that hepcidin expression is up-regulated in many cancers, including advanced stages of colorectal cancer (CRC), one of the most frequent neoplasms and a major cause of cancer death worldwide. Accumulating evidence suggests that hepcidin controls multiple steps that sustain CRC cell growth, survival, and diffusion. Hence, the inhibition of CRC-derived hepcidin helps attenuate CRC progression. In this review article, we review the data supporting the regulatory effects of hepcidin on colon tumorigenesis and discuss the potential impact of hepcidin inhibition on the evolutive phases of CRC.

## 1. Introduction

Colorectal cancer (CRC) is one of the most frequent neoplasms and the second cause of cancer-related mortality worldwide [1]. According to Global Cancer Observatory (GLOBOCAN) estimates, in 2020, there were more than 1.9 million new cases of CRC, which accounted for nearly one million deaths globally [2]. Although, in the last decades, screening programs (i.e., fecal immunochemical testing and colonoscopy or sigmoidoscopy) have facilitated the detection of endoscopically removable precancerous polyps and diagnosis of early CRC, nearly 20% of CRCs are diagnosed when they are already metastatic. The location of neoplasia influences the behavior and response to treatment because right-sided CRCs are often detected in advanced stages; they are less responsive to certain targeted therapies (e.g., anti-epidermal growth factor receptor antibodies) and, therefore, associated with worse prognosis [3,4]. Moreover, metastatic CRC with microsatellite instability (MSI) responds quite well to immune checkpoint inhibitors (ICIs) [5], while microsatellite stable (MSS) metastatic CRCs, which are more than 80% of all CRCs, are unresponsive to ICIs [6], and the 5-year overall survival (OS) rate of this latter group of patients is lower than 20% after standard chemotherapy with or without drugs targeting specific pathways (e.g., antiangiogenic drugs) [7,8]. These observations suggest the need for further studies aimed at identifying novel therapeutic targets for metastatic CRC. Classically known as a regulator of iron balance and mainly produced in the liver, hepcidin is a peptide synthesized by cancer cells in many organs, including CRC cells. Accumulating evidence indicates that CRC cell-derived hepcidin can control multiple pathways that sustain CRC cell growth and metastasis. In the present review article, we discuss the available data supporting the role of hepcidin in colon tumorigenesis and discuss how hepcidin inhibitors can help treat CRC patients in the metastatic setting with particular regard to the impact of hepcidin modulation on immunotherapeutic outcomes.

## 2. Hepcidin Expression and Function

Hepcidin is synthesized as pre-prohepcidin that is cleaved to produce the 60 amino-acid peptide prohepcidin. Prohepcidin is then cleaved by furin into two isoforms, the mature hepcidin of 25 amino acids and a peptide the function of which is unknown. The mature hepcidin binds to ferroportin (FPN) 1, thereby leading to FPN1 internalization and proteolysis, predominantly in lysosomes, with the downstream effect of inhibiting the efflux of iron from the intracellular compartment of iron-storing cells to the systemic circulation [9,10]. As a consequence, in the conditions characterized by low hepcidin production, there is a high expression of FPN1 that facilitates the export of iron from iron-transporting cells/tissues to extracellular fluids. This is seen, for example, in both humans and mice with genetic hepcidin loss, which causes severe forms of iron overload [11,12]. In contrast, when the levels of hepcidin are high, FPN1 tends to disappear from cell membranes, and hence, the export of iron to plasma ceases [13,14,15].

FPN1 is highly expressed by periportal hepatocytes and macrophages in the spleen and the liver, where these cells recycle iron from senescent erythrocytes. FPN1 is also expressed by duodenal enterocytes. Accordingly, hepcidin is mainly produced by hepatocytes and, to a lesser degree, by macrophages and epithelial cells [16]. Moreover, hepcidin is expressed by human adipose tissue [17]. In the liver, hepcidin plays a role in addition to controlling iron cellular levels, as the peptide has been implicated in the intercellular communication between hepatocytes and hepatic stellate cells (HSCs). Specifically, by promoting FPN1 degradation on the surface of HSCs, hepcidin inhibits the activation of HSCs, thereby suppressing the production of extracellular matrix and inflammatory cytokines and eventually improving liver fibrosis [18].

**Figure 1:**
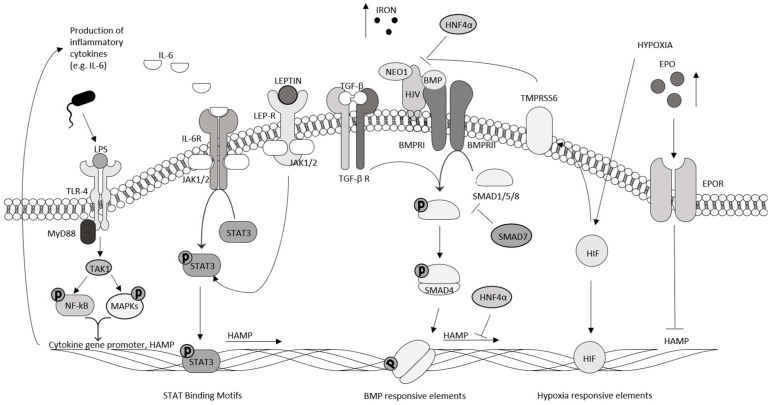
Main pathways involved in the regulation of hepcidin levels. The figure shows the main pathways regulating hepcidin expression in response to various physiological and environmental stimuli, such as iron levels, inflammation, and hypoxia. In conditions characterized by high iron levels, the bone morphogenic protein (BMP)/Sma and mothers against decapentaplegic homolog (SMAD) pathway is activated by binding of BMP proteins to receptors in cooperation with the co-receptors hemojuvelin (HJV) and neogenin (NEO1). This process leads to the phosphorylation of the proteins Smad1/5/8, which form a complex with Smad4 that moves into the nucleus to activate hepcidin gene transcription. Hepcidin can also be induced by transforming growth factor beta (TGF-β 1) through a mechanism mediated by Smad1/5/8. In inflammatory conditions, cytokines, such as interleukin (IL)-6, activate the Janus kinase (JAK)/signal transducers and activators of the transcription (STAT) pathway, leading to hepcidin induction. Activation of toll-like receptor-4 (TLR4) by bacterial lipopolysaccharide (LPS) stimulates the production of hepcidin through the transcription factors of nuclear factor kappa-light-chain-enhancer of activated B cells (NF-κB) and mitogen-activated protein kinases (MAPKs). An additional stimulus for the induction of hepcidin is leptin, which is produced abundantly in obese individuals. In contrast, in conditions marked by hypoxia, the hypoxia inducible factor (HIF) pathway inhibits hepcidin expression by increasing iron availability for erythropoiesis through the production of erythropoietin (EPO).

Hepcidin gene expression is tightly controlled by various factors, including iron load and inflammatory molecules (Figure 1). Low iron levels and increased erythropoiesis reduce hepcidin expression, which is followed by increased iron absorption and efflux of iron from storing cells to circulation. In contrast, high iron levels up-regulate hepcidin expression, thereby reducing iron absorption and keeping iron in hepatocytes and macrophages [19]. Hepatic hepcidin synthesis is also regulated by the bone morphogenetic protein (BMP) pathway. Specifically, upon the binding of iron-induced BMP2/4/6/9 proteins to the BMP receptor expressed on hepatocytes, the activated BMP receptor cooperates with two transmembrane co-receptors, Hemojuvelin and Neogenin, to induce the phosphorylation of Smad1/5/8, which interact with Smad4, thereby allowing the complex to move to the nucleus and enhance hepcidin promoter activity [20,21]. Hepcidin can also be induced by TGF-β1 through a mechanism involving Smad1/5/8 rather than the classical Smad2/3 pathway [22]. Consistently, the hepatocyte-specific knockout of Smad7, an inhibitor of TGF-β1 and BMP signaling, leads to an increased phosphorylation of Smad1/5/8 and up-regulation of hepcidin, while an over-expression of Smad7 in primary murine hepatocytes reduces hepcidin production [23,24]. Hepcidin expression is also inhibited by the hepatocyte nuclear factor-4α, another negative regulator of BMP signaling [25], as well as by both hypoxia and the hypoxia-inducible factor, through increases in erythropoietin synthesis and matriptase-2 [26,27]. Another level of control of hepcidin expression relies on inflammatory molecules [e.g., interleukin (IL)-6], which trigger the Janus kinase (Jak)/signal transducer and activator of the transcription (Stat) 3 pathway. This pathway is crucial during infections for reducing serum iron levels and, hence, limiting the survival and replication of microorganisms [28]. Hepatocyte-derived hepcidin can be enhanced by the lipopolysaccharide (LPS)-induced activation of toll-like receptor-4 expressed on hepatocytes through a mechanism dependent on NF-kB, c-Jun N-terminal kinase, and activator protein-1 [29]. LPS can also increase hepcidin production by primarily facilitating Smad signaling [30]. Additional stimuli for the induction of hepcidin include leptin, which is highly produced in obese individuals [31,32], and acute-phase proteins released in chronic pathologies, such as cardiovascular diseases and cancer [27].

Bone morphogenetic protein receptor I (BMPRI), bone morphogenetic protein receptor II (BMPRII), erythropoietin receptor (EPO-R), hepatocyte nuclear factor-4α (HNF4α), leptin receptor (LEP-R), myeloid differentiation primary response 88 (MyD88), TGF-β-activated kinase 1 (TAK1), matriptase-2 (TMPRSS6), and hepcidin-antimicrobial peptide (HAMP) are displayed in the image, which was designed with PowerPoint software.

## 3. Hepcidin and Cancers

Since the dysregulation of iron homeostasis has been documented and associated with worse outcomes in many cancers [33], many researchers have assessed the expression and role of hepcidin in both pre-malignant conditions and malignancies. A reduced hepatic expression of hepcidin was documented in patients with cirrhosis, a chronic liver disorder that may eventually lead to hepatocellular carcinoma (HCC) [34]. In HCC tissues, the hepcidin gene is repressed, probably as a result of promoter hypermethylation [35]. In contrast, a high expression of hepcidin has been seen in non-small cell lung cancer cells, and functional studies showed that hepcidin activates NF-kB in such cells, thus promoting proliferation, invasion, and migration [36]. In lung cancer patients, there is a significant association between hepcidin expression and metastasis, and the cancer tissue-associated hepcidin expression predicts an unfavorable prognosis [37]. Hepcidin is highly expressed in breast cancer, where it is supposed to contribute to the development of the malignant phenotype and resistance to cytostatic drugs [38,39].

Single nucleotide polymorphisms of hepcidin and hepcidin-regulating genes (i.e., transferrin receptor (TFR) 1 and 2, hemojuvelin, and BMP6 have been associated with the risk of pancreatic ductal adenocarcinoma [40], and high hepcidin expression in pancreatic cancer tissue is a prognostic factor of OS in patients with such a malignancy [41]. High hepcidin expression has been documented in kidney and testicular cancers, and the level of hepcidin is a prognostic indicator in male patients with genitourinary system cancers, especially kidney renal clear cell carcinoma [42]. Hepcidin has also been implicated in the development of prostate cancer, given its ability to enhance the proliferation, survival, and migration capacities of prostatic cancer cells [43,44]. High serum hepcidin levels occur in children and adults with acute leukemia independently of the phase of the disease [45].

However, these data indicate that the dysregulation of hepcidin expression may play a role in the carcinogenetic process in various organs. In this context, it is also noteworthy that over-expression of hepcidin in cancer patients contributes to anemia because of the reduced availability of iron for erythropoiesis secondary to the sequestration of iron in cells, mainly in macrophages [46]. On the other hand, this function of hepcidin can be useful for negatively controlling erythrocytosis and alleviating symptoms in other neoplastic diseases. A classic example is provided by polycythemia vera, a myeloproliferative neoplasm that is characterized by erythrocytosis, increased leukocyte and platelet counts, and enhanced risk of thromboembolic events [47]. Indeed, preclinical studies and recent clinical trials with hepcidin mimetics indicate that enhancing the hepcidin activity in patients with polycythemia vera is effective in controlling erythrocytosis [48,49].

### 3.1. Expression and Role of Hepcidin in Colorectal Cancer

Cellular iron can influence several pathways involved in the development of CRC [50], and in CRC tissue, there is an over-production of factors (i.e., IL-6, leptin) implicated in the regulation of hepcidin synthesis [51,52]. These observations boosted research aimed to assess the expression and function of hepcidin in CRC. In an initial study, Sornjaithe and colleagues showed that hepcidin RNA expression was more pronounced in CRC samples than in matched normal colonic samples, and the treatment of the CRC cell line HT29 with hepcidin increased cell survival. However, no effect was seen in HCT-116, SW620, and SW480 cell survival exposed to hepcidin. These differences probably reflect the high expression of FPN1 mRNA in HT29 cells as compared to expression in the other cell lines [53]. More recently, by using mice with intestinal epithelial cell loss of hepcidin, Schwartz and colleagues showed that hepcidin is essential for degrading FPN1 and sequestering iron into the cells, thereby facilitating the production of nucleotides and CRC cell growth [54]. Along the same lines are our data showing that hepcidin RNA and protein are over-expressed in CRC tissues and cancer cells are the major source of hepcidin in the tumor area [55]. Moreover, an evaluation of bioinformatics databases confirmed the enhanced hepcidin RNA expression in CRC samples regardless of the clinical features of the patients [55]. We also showed that cytokines activating Stat3, like IL-6, IL-21, and IL-23, enhance the production of hepcidin in normal colonic epithelial cells, while the inhibition of Stat3 in CRC cells reduces hepcidin synthesis [55]. Additionally, in human CRC tissue, Stat3 co-localizes with hepcidin [55], further supporting the hypothesis that induction of hepcidin in CRC cells can, at least in part, rely on Stat3 activation.

In the last decades, several studies have convincingly shown that various cell types present within the CRC microenvironment can inhibit CRC cell behavior and influence the outcome of chemotherapy and immunotherapy [56,57]. One such cell type is the regulatory macrophage, which inhibits effector T-cell functions [58]. Notably, in CRC samples, hepcidin expression correlates with the expression of CD206 and IL-10, two markers of regulatory macrophages [55]. Moreover, the treatment of circulating monocytes with hepcidin enhances the fractions of CD206- and IL-10-expressing cells [55], suggesting a role for hepcidin in the differentiation of regulatory macrophages. However, these findings help delineate a scenario in which high production of hepcidin by cancer cells, and perhaps additional cell types within the tumor niche, contributes to generating a microenvironment that dampens antitumor immunity and promotes cancer progression. Consistent with this hypothesis is the demonstration that hepcidin RNA expression is significantly higher in patients with metastatic CRC than in those without metastasis [55]. Moreover, hepcidin expression correlates with several markers of epithelial-to-mesenchymal transition, a phenomenon involved in the cancer metastatic process, and the inhibition of hepcidin in CRC cells reduces such markers [55].

### 3.2. Hepcidin Is a Negative Regulator of Antigen-Specific Immunity Against Colorectal Cancer Cells

**Figure 2:**
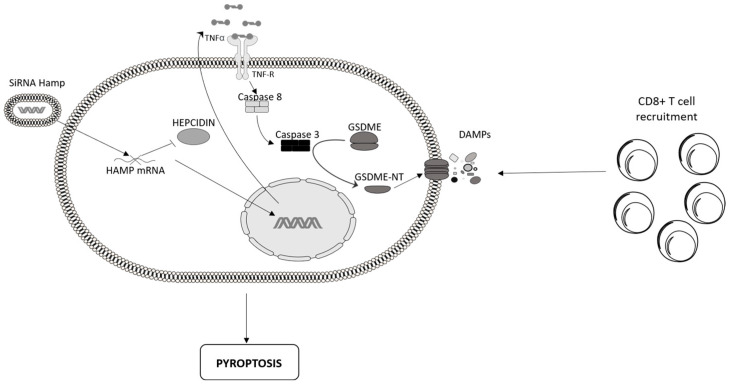
Hepcidin silencing induces pyroptosis in colorectal cancer cells. Silencing hepcidin via small interfering RNA (siRNA) in colorectal cancer cells induces the production of tumor necrosis factor α (TNF-α), which promotes the activation of caspase-3 and -8. The cleavage of Gasdermin E (GSDM E), mediated by activated caspases, produces a Gasdermin E N-Terminale (GSDM E-NT) fragment with the downstream effect of inducing pyroptosis, release of damage-associated molecular patterns (DAMPs), and consequently recruitment of CD8+ T lymphocytes into the tumor tissue. Tumor necrosis factor receptor (TNF-R) and hepcidin-antimicrobial peptide (HAMP) are shown in the image, which was designed with PowerPoint software.

Antigen-specific immune cells (e.g., CD8+ T cells) can kill malignant cells, a process termed cancer immunosurveillance. Nonetheless, as stated above, cancer cells can produce several molecules that target immunosuppressive cells within the tumor microenvironment (e.g., regulatory macrophages, myeloid suppressor cells), thereby triggering a mechanism that attenuates the anti-cancer immunity and ultimately promotes cancer progression and metastasis [59,60,61]. To further explore the effect of CRC cell-derived hepcidin on the ongoing immune response, we silenced hepcidin in HCT116 cells and evaluated the effect of hepcidin inhibition on anti-cancer immunity. Hepcidin silencing was accompanied by morphological changes in cells (i.e., ballooning cell membranes), enhanced SYTOX green uptake, and secretion of high mobility group protein 1, all features consistent with the induction of pyroptosis, a type of immunogenic lytic cellular suicide [62]. Moreover, hepcidin silencing promoted the cleavage of gasdermin (GSDM) E, a protein that has recently been identified as a critical executor of chemotherapy drug-induced pyroptosis [63] (Figure 2). GSDM E silencing largely reduced pyroptosis in hepcidin-deficient cells. GSDM E is ubiquitously expressed in most normal human cells, while it is epigenetically suppressed by promoter deoxyribonucleic acid methylation in several cancers, including CRC [64]. We profiled other CRC cells and found that hepcidin silencing induces pyroptosis in CT26 cells, which express GSDM E but not in the GSDM E-negative SW480. Interestingly, the induction of GSDM E in SW480 cells by the DNA methyltransferase inhibitor decitabine was accompanied by pyroptosis following hepcidin silencing [62].

In human cells, the cleavage of GSDM E, mediated by activated caspases, produces a GSDM E-NT fragment, thus switching the cell death mode from apoptosis to pyroptotic cell death [65]. Among the various caspases involved in GSDM E cleavage, only caspase-8 was activated by hepcidin silencing, and this event was mediated by TNF, as supported by the demonstration that TNF expression was up-regulated by hepcidin silencing and neutralization of TNF in hepcidin-silenced HCT116 cell cultures reduced the cleavage of GSDM E and induction of pyroptosis [62] (Figure 2).

To translate these observations to in vivo models of CRC, we engrafted mice with wild-type (WT) or hepcidin-silenced CT26 cells and observed that hepcidin deficiency reduced the growth of tumors. Hepcidin-silenced cell-derived tumors exhibited a marked infiltration of perforin and granzyme B-expressing CD8+ cells, the depletion of which reverted the protective effect of hepcidin deficiency on the growth of tumors [62] (Figure 2). These findings confirm and expand on data from recent studies showing that the induction of death in GSDM E-expressing cancers either by intrinsic stresses or extrinsic challenges promotes the recruitment of NK and CD8+ T lymphocytes into the tumor microenvironment and suppresses tumor growth [66]. Implanted hepcidin-silenced CT26 cells protected mice from challenge with WT CT26 cells. Protection by vaccination, the gold standard for immunogenic cell death, was specific, as no protection was seen when mice were re-challenged with TS/A, a mammary cancer cell line that expresses a repertoire of antigens different from those of CT26. This phenomenon persisted over time and did not require additional antitumor therapies [62].

As outlined above, most metastatic CRCs are mismatch repair proficient and/or MSS and, therefore, unresponsive to PD-L1 and PD-1 ICIs [67]. PD-1, which is mainly expressed by lymphocytes, NK cells, and myeloid-derived suppressor cells, binds to its ligands (i.e., PD-L1/2) expressed on antigen-presenting cells and cancer cells [68]. Because the interaction between PD-1 and PD-L1/2 inhibits T-cell activation and cytotoxic activity and transforms T effector cells into regulatory T cells, a blockade of the PD-1/PD-L1/2 pathway enhances T-cell-dependent antitumor activity [69,70]. Since pyroptosis enhances the response of cancer cells to ICIs [71], we finally tested the possibility that hepcidin silencing can make CRC cells with MSS (i.e., CT26) more responsive to αPD-1. Hepcidin silencing in combination with αPD-1 reduced the CT26-derived tumor growth significantly and enhanced the number of tumor-infiltrating TNF-secreting CD8+ T cells as compared to controls [62].

## 4. Discussion

The findings illustrated in this article support the role of hepcidin in CRC not only as a potential inducer of anemia but also as a positive regulator of several pathways that sustain CRC cell growth, survival, and diffusion. The present evidence indicates that CRC cells produce hepcidin, which, in a paracrine or autocrine manner, stimulates CRC cell proliferation and blocks the apoptotic machinery. It is, however, likely that the cancer cell-secreted hepcidin can target additional cell types within the tumor microenvironment (e.g., regulatory macrophages), with the downstream effect of enhancing the carcinogenetic process. Inhibition of hepcidin in CRC cells is associated with the induction of pyroptosis and the release of a variety of molecules that stimulate the recruitment and activation of immune cells, including a pool of antigen-specific cytotoxic cells. Notably, hepcidin inhibition increases the response of mice bearing CT26-derived tumors to PD-1 blockers [62], raising the possibility that reducing hepcidin levels in CRC cells increases the immunogenicity of the tumor microenvironment, converting cold tumors to hot tumors. Altogether, these data suggest that the blockade of hepcidin can help attenuate colon carcinogenesis, and this can have major relevance in metastatic settings as hepcidin is mainly produced during the later stages of the disease [55]. However, inhibiting the hepcidin effects during the evolutive phases of CRC cannot be as simple as one can theoretically anticipate. The attempts to directly down-regulate hepcidin expression by using either humanized monoclonal antibodies or short interfering RNA have not been followed by commercial developments despite the initial studies documenting that such treatments were well-tolerated [72]. Other strategies to block hepcidin include PRS-080, an anticalin that binds to and decreases hepcidin protein levels, and drugs inhibiting pathways involved in hepcidin synthesis (e.g., BMP6) or modulating the hepcidin–ferroportin axis [73,74,75]. For a detailed description of hepcidin inhibitors, the reader is directed toward recent reviews [75,76]. In this context, it is, however, noteworthy that further experimentation in preclinical models of CRC is needed to assess the therapeutic effect of such compounds.

Little is known about the basic mechanisms by which hepcidin controls the different phases of colon tumorigenesis, even though it is conceivable that most functions rely on the control of iron balance and are, at least in part, linked to the ability of the hormone to activate Stat3, a transcription factor controlling multiple steps in CRC [77]. Similarly, the factors involved in the control of CRC tissue-associated hepcidin expression remain largely unknown, but recent studies have raised the possibility that cells present in the tumor microenvironment can influence hepcidin production. For example, it has been demonstrated that tumor-associated fibroblasts are a powerful stimulus for the induction of breast cancer cell-derived hepcidin, and this induction relies on the fibroblast-dependent secretion of IL-6 [78]. It is also noteworthy that hepcidin-induced Stat3 activation can result in enhanced hepcidin synthesis, thereby triggering a positive feedback loop that amplifies hepcidin production [55]. We have recently shown that serum hepcidin levels are an independent risk factor for the overall survival in metastatic CRC patients undergoing standard first-line treatment [79], but further, larger studies are needed to confirm such findings.

## 5. Conclusions

The available evidence indicates that hepcidin is highly expressed in CRC, particularly in the later stages of the neoplasia, and suggests that blockers of hepcidin production/function can represent a novel way to treat patients with advanced CRC.

## References

[1] Bray F., Ferlay J., Soerjomataram I., Siegel R.L., Torre L.A., Jemal A. (2018). Global Cancer Statistics 2018: GLOBOCAN Estimates of Incidence and Mortality Worldwide for 36 Cancers in 185 Countries. CA Cancer J. Clin..

[2] Morgan E., Arnold M., Gini A., Lorenzoni V., Cabasag C.J., Laversanne M., Vignat J., Ferlay J., Murphy N., Bray F. (2023). Global Burden of Colorectal Cancer in 2020 and 2040: Incidence and Mortality Estimates from GLOBOCAN. Gut.

[3] Lee G.H., Malietzis G., Askari A., Bernardo D., Al-Hassi H.O., Clark S.K. (2015). Is Right-Sided Colon Cancer Different to Left-Sided Colorectal Cancer?—A Systematic Review. Eur. J. Surg. Oncol. (EJSO).

[4] Yahagi M., Okabayashi K., Hasegawa H., Tsuruta M., Kitagawa Y. (2016). The Worse Prognosis of Right-Sided Compared with Left-Sided Colon Cancers: A Systematic Review and Meta-Analysis. J. Gastrointest. Surg..

[5] Andre T., Amonkar M., Norquist J.M., Shiu K.-K., Kim T.W., Jensen B.V., Jensen L.H., Punt C.J.A., Smith D., Garcia-Carbonero R. (2021). Health-Related Quality of Life in Patients with Microsatellite Instability-High or Mismatch Repair Deficient Metastatic Colorectal Cancer Treated with First-Line Pembrolizumab versus Chemotherapy (KEYNOTE-177): An Open-Label, Randomised, Phase 3 Trial. Lancet Oncol..

[6] Koopman M., Kortman G.a.M., Mekenkamp L., Ligtenberg M.J.L., Hoogerbrugge N., Antonini N.F., Punt C.J.A., van Krieken J.H.J.M. (2009). Deficient Mismatch Repair System in Patients with Sporadic Advanced Colorectal Cancer. Br. J. Cancer.

[7] Li J., Qin S., Xu R., Yau T.C.C., Ma B., Pan H., Xu J., Bai Y., Chi Y., Wang L. (2015). Regorafenib plus Best Supportive Care versus Placebo plus Best Supportive Care in Asian Patients with Previously Treated Metastatic Colorectal Cancer (CONCUR): A Randomised, Double-Blind, Placebo-Controlled, Phase 3 Trial. Lancet Oncol..

[8] Li J., Qin S., Xu R.-H., Shen L., Xu J., Bai Y., Yang L., Deng Y., Chen Z., Zhong H. (2018). Effect of Fruquintinib vs. Placebo on Overall Survival in Patients with Previously Treated Metastatic Colorectal Cancer: The FRESCO Randomized Clinical Trial. JAMA.

[9] Hunter H.N., Fulton D.B., Ganz T., Vogel H.J. (2002). The Solution Structure of Human Hepcidin, a Peptide Hormone with Antimicrobial Activity That Is Involved in Iron Uptake and Hereditary Hemochromatosis. J. Biol. Chem..

[10] Nemeth E., Tuttle M.S., Powelson J., Vaughn M.B., Donovan A., Ward D.M., Ganz T., Kaplan J. (2004). Hepcidin Regulates Cellular Iron Efflux by Binding to Ferroportin and Inducing Its Internalization. Science.

[11] Roetto A., Papanikolaou G., Politou M., Alberti F., Girelli D., Christakis J., Loukopoulos D., Camaschella C. (2003). Mutant Antimicrobial Peptide Hepcidin Is Associated with Severe Juvenile Hemochromatosis. Nat. Genet..

[12] Lesbordes-Brion J.-C., Viatte L., Bennoun M., Lou D.-Q., Ramey G., Houbron C., Hamard G., Kahn A., Vaulont S. (2006). Targeted Disruption of the Hepcidin 1 Gene Results in Severe Hemochromatosis. Blood.

[13] Nicolas G., Bennoun M., Porteu A., Mativet S., Beaumont C., Grandchamp B., Sirito M., Sawadogo M., Kahn A., Vaulont S. (2002). Severe Iron Deficiency Anemia in Transgenic Mice Expressing Liver Hepcidin. Proc. Natl. Acad. Sci. USA.

[14] Rivera S., Nemeth E., Gabayan V., Lopez M.A., Farshidi D., Ganz T. (2005). Synthetic Hepcidin Causes Rapid Dose-Dependent Hypoferremia and Is Concentrated in Ferroportin-Containing Organs. Blood.

[15] Laftah A.H., Ramesh B., Simpson R.J., Solanky N., Bahram S., Schümann K., Debnam E.S., Srai S.K.S. (2004). Effect of Hepcidin on Intestinal Iron Absorption in Mice. Blood.

[16] Drakesmith H., Nemeth E., Ganz T. (2015). Ironing out Ferroportin. Cell Metab..

[17] Bekri S., Gual P., Anty R., Luciani N., Dahman M., Ramesh B., Iannelli A., Staccini–Myx A., Casanova D., Ben Amor I. (2006). Increased Adipose Tissue Expression of Hepcidin in Severe Obesity Is Independent from Diabetes and NASH. Gastroenterology.

[18] Han C.Y., Koo J.H., Kim S.H., Gardenghi S., Rivella S., Strnad P., Hwang S.J., Kim S.G. (2016). Hepcidin Inhibits Smad3 Phosphorylation in Hepatic Stellate Cells by Impeding Ferroportin-Mediated Regulation of Akt. Nat. Commun..

[19] Ganz T., Nemeth E. (2012). Hepcidin and Iron Homeostasis. Biochim. Biophys. Acta (BBA)-Mol. Cell Res..

[20] Xia Y., Babitt J.L., Sidis Y., Chung R.T., Lin H.Y. (2008). Hemojuvelin Regulates Hepcidin Expression via a Selective Subset of BMP Ligands and Receptors Independently of Neogenin. Blood.

[21] Xiao X., Alfaro-Magallanes V.M., Babitt J.L. (2020). Bone Morphogenic Proteins in Iron Homeostasis. Bone.

[22] Chen Q., Wang L., Ma Y., Wu X., Jin L., Yu F. (2014). Increased Hepcidin Expression in Non-small Cell Lung Cancer Tissue and Serum Is Associated with Clinical Stage. Thorac. Cancer.

[23] An P., Wang H., Wu Q., Wang J., Xia Z., He X., Wang X., Chen Y., Min J., Wang F. (2018). Smad7 Deficiency Decreases Iron and Haemoglobin through Hepcidin Up-regulation by Multilayer Compensatory Mechanisms. J. Cell. Mol. Med..

[24] Mleczko-Sanecka K., Casanovas G., Ragab A., Breitkopf K., Müller A., Boutros M., Dooley S., Hentze M.W., Muckenthaler M.U. (2010). SMAD7 Controls Iron Metabolism as a Potent Inhibitor of Hepcidin Expression. Blood.

[25] Shi W., Wang H., Zheng X., Jiang X., Xu Z., Shen H., Li M. (2017). HNF-4alpha Negatively Regulates Hepcidin Expression Through BMPR1A in HepG2 Cells. Biol. Trace Elem. Res..

[26] Enns C.A., Weiskopf T., Zhang R.H., Wu J., Jue S., Kawaguchi M., Kataoka H., Zhang A.-S. (2023). Matriptase-2 Regulates Iron Homeostasis Primarily by Setting the Basal Levels of Hepatic Hepcidin Expression through a Nonproteolytic Mechanism. J. Biol. Chem..

[27] Formica V., Riondino S., Morelli C., Guerriero S., D’Amore F., Di Grazia A., Del Vecchio Blanco G., Sica G., Arkenau H.-T., Monteleone G. (2023). HIF2α, Hepcidin and Their Crosstalk as Tumour-Promoting Signalling. Br. J. Cancer.

[28] Wang C.-Y., Babitt J.L. (2016). Hepcidin Regulation in the Anemia of Inflammation. Curr. Opin. Hematol..

[29] Lee Y.-S., Kim Y.-H., Jung Y.S., Kim K.-S., Kim D.-K., Na S.-Y., Lee J.-M., Lee C.-H., Choi H.-S. (2017). Hepatocyte Toll-like Receptor 4 Mediates Lipopolysaccharide-Induced Hepcidin Expression. Exp. Mol. Med..

[30] Kowdley K.V., Gochanour E.M., Sundaram V., Shah R.A., Handa P. (2021). Hepcidin Signaling in Health and Disease: Ironing Out the Details. Hepatol. Commun..

[31] Yamamoto K., Kuragano T., Kimura T., Nanami M., Hasuike Y., Nakanishi T. (2018). Interplay of Adipocyte and Hepatocyte: Leptin Upregulates Hepcidin. Biochem. Biophys. Res. Commun..

[32] Chung B., Matak P., McKie A.T., Sharp P. (2007). Leptin Increases the Expression of the Iron Regulatory Hormone Hepcidin in HuH7 Human Hepatoma Cells. J. Nutr..

[33] Ru Q., Li Y., Chen L., Wu Y., Min J., Wang F. (2024). Iron Homeostasis and Ferroptosis in Human Diseases: Mechanisms and Therapeutic Prospects. Signal Transduct. Target. Ther..

[34] Joachim J.H., Mehta K.J. (2022). Hepcidin in Hepatocellular Carcinoma. Br. J. Cancer.

[35] Udali S., Castagna A., Corbella M., Ruzzenente A., Moruzzi S., Mazzi F., Campagnaro T., De Santis D., Franceschi A., Pattini P. (2018). Hepcidin and DNA Promoter Methylation in Hepatocellular Carcinoma. Eur. J. Clin. Investig..

[36] Li Z., Liu J., Wang P., Zhang B., He G., Yang L. (2024). HAMP Predicts a Pivotal Role in Modulating the Malignant Behaviors of Non-Small Cell Lung Cancer Cells. Aging.

[37] Fan Y., Liu B., Chen F., Song Z., Han B., Meng Y., Hou J., Cao P., Chang Y., Tan K. (2021). Hepcidin Upregulation in Lung Cancer: A Potential Therapeutic Target Associated with Immune Infiltration. Front. Immunol..

[38] Pan X., Lu Y., Cheng X., Wang J. (2016). Hepcidin and Ferroportin Expression in Breast Cancer Tissue and Serum and Their Relationship with Anemia. Curr. Oncol..

[39] Yalovenko T.M., Todor I.M., Lukianova N.Y., Chekhun V.F. (2016). Hepcidin as a possible marker in determination of malignancy degree and sensitivity of breast cancer cells to cytostatic drugs. Exp. Onc..

[40] Julián-Serrano S., Yuan F., Wheeler W., Benyamin B., Machiela M.J., Arslan A.A., Beane-Freeman L.E., Bracci P.M., Duell E.J., Du M. (2021). Hepcidin-Regulating Iron Metabolism Genes and Pancreatic Ductal Adenocarcinoma: A Pathway Analysis of Genome-Wide Association Studies. Am. J. Clin. Nutr..

[41] Toshiyama R., Konno M., Eguchi H., Asai A., Noda T., Koseki J., Asukai K., Ohashi T., Matsushita K., Iwagami Y. Association of Iron Metabolic Enzyme Hepcidin Expression Levels with the Prognosis of Patients with Pancreatic Cancer. Oncol. Lett..

[42] Wang X., Shi Q., Gong P., Zhou C., Cao Y. (2022). An Integrated Systematic Analysis and the Clinical Significance of Hepcidin in Common Malignancies of the Male Genitourinary System. Front. Genet..

[43] Zhao B., Li R., Cheng G., Li Z., Zhang Z., Li J., Zhang G., Bi C., Hu C., Yang L. (2018). Role of Hepcidin and Iron Metabolism in the Onset of Prostate Cancer. Oncol. Lett..

[44] Tesfay L., Clausen K.A., Kim J.W., Hegde P., Wang X., Miller L.D., Deng Z., Blanchette N., Arvedson T., Miranti C.K. (2015). Hepcidin Regulation in Prostate and Its Disruption in Prostate Cancer. Cancer Res..

[45] Słomka A., Łęcka M., Styczyński J. (2022). Hepcidin in Children and Adults with Acute Leukemia or Undergoing Hematopoietic Cell Transplantation: A Systematic Review. Cancers.

[46] Ganz T. (2019). Anemia of Inflammation. N. Engl. J. Med..

[47] Tefferi A., Vannucchi A.M., Barbui T. (2021). Polycythemia Vera: Historical Oversights, Diagnostic Details, and Therapeutic Views. Leukemia.

[48] Casu C., Nemeth E., Rivella S. (2018). Hepcidin Agonists as Therapeutic Tools. Blood.

[49] Kremyanskaya M., Kuykendall A.T., Pemmaraju N., Ritchie E.K., Gotlib J., Gerds A., Palmer J., Pettit K., Nath U.K., Yacoub A. (2024). Rusfertide, a Hepcidin Mimetic, for Control of Erythrocytosis in Polycythemia Vera. N. Engl. J. Med..

[50] Padmanabhan H., Brookes M.J., Iqbal T. (2015). Iron and Colorectal Cancer: Evidence from in Vitro and Animal Studies. Nutr. Rev..

[51] Jones A.N., Scheurlen K.M., Macleod A., Simon H.L., Galandiuk S. (2024). Obesity and Inflammatory Factors in the Progression of Early-Onset Colorectal Cancer. Cancers.

[52] Wang S.-W., Sun Y.-M. (2014). The IL-6/JAK/STAT3 Pathway: Potential Therapeutic Strategies in Treating Colorectal Cancer. Int. J. Oncol..

[53] Sornjai W., Nguyen Van Long F., Pion N., Pasquer A., Saurin J.-C., Marcel V., Diaz J.J., Mertani H.C., Smith D.R. (2020). Iron and Hepcidin Mediate Human Colorectal Cancer Cell Growth. Chem. Biol. Interact..

[54] Schwartz A.J., Goyert J.W., Solanki S., Kerk S.A., Chen B., Castillo C., Hsu P.P., Do B.T., Singhal R., Dame M.K. (2021). Hepcidin Sequesters Iron to Sustain Nucleotide Metabolism and Mitochondrial Function in Colorectal Cancer Epithelial Cells. Nat. Metab..

[55] Di Grazia A., Di Fusco D., Franzè E., Colella M., Strimpakos G., Salvatori S., Formica V., Laudisi F., Maresca C., Colantoni A. (2022). Hepcidin Upregulation in Colorectal Cancer Associates with Accumulation of Regulatory Macrophages and Epithelial–Mesenchymal Transition and Correlates with Progression of the Disease. Cancers.

[56] Monteleone G., Maresca C., Colella M., Pacifico T., Congiu D., Troncone E., Marafini I. (2022). Targeting IL-34/MCSF-1R Axis in Colon Cancer. Front. Immunol..

[57] Quail D.F., Joyce J.A. (2013). Microenvironmental Regulation of Tumor Progression and Metastasis. Nat. Med..

[58] Biswas S.K., Mantovani A. (2010). Macrophage Plasticity and Interaction with Lymphocyte Subsets: Cancer as a Paradigm. Nat. Immunol..

[59] Gatti R.A., Good R.A. (1971). Occurrence of Malignancy in Immunodeficiency Diseases: A Literature Review. Cancer.

[60] Li K., Shi H., Zhang B., Ou X., Ma Q., Chen Y., Shu P., Li D., Wang Y. (2021). Myeloid-Derived Suppressor Cells as Immunosuppressive Regulators and Therapeutic Targets in Cancer. Signal Transduct. Target. Ther..

[61] Kono K., Mimura K., Kiessling R. (2013). Immunogenic Tumor Cell Death Induced by Chemoradiotherapy: Molecular Mechanisms and a Clinical Translation. Cell Death Dis..

[62] Di Grazia A., Franzè E., Frascatani R., Laudisi F., Pacifico T., Tomassini L., Di Fusco D., Formica V., Sica G., Stolfi C. (2024). Targeting Hepcidin in Colorectal Cancer Triggers a TNF-Dependent-Gasdermin E-Driven Immunogenic Cell Death Response. Exp. Hematol. Oncol..

[63] Mei Z., Chen X., Chen Y., Su X., Lv S., Wei S. (2023). Improved Antitumor Immunity of Chemotherapy in OSCC Treatment by Gasdermin-E Mediated Pyroptosis. Apoptosis.

[64] Liu X., Xia S., Zhang Z., Wu H., Lieberman J. (2021). Channelling Inflammation: Gasdermins in Physiology and Disease. Nat. Rev. Drug Discov..

[65] Jiang M., Qi L., Li L., Li Y. (2020). The Caspase-3/GSDME Signal Pathway as a Switch between Apoptosis and Pyroptosis in Cancer. Cell Death Discov..

[66] Zhang Z., Zhang Y., Xia S., Kong Q., Li S., Liu X., Junqueira C., Meza-Sosa K.F., Mok T.M.Y., Ansara J. (2020). Gasdermin E Suppresses Tumour Growth by Activating Anti-Tumour Immunity. Nature.

[67] Lin K.X., Istl A.C., Quan D., Skaro A., Tang E., Zheng X. (2023). PD-1 and PD-L1 Inhibitors in Cold Colorectal Cancer: Challenges and Strategies. Cancer Immunol. Immunother..

[68] Dong Y., Sun Q., Zhang X. (2017). PD-1 and Its Ligands Are Important Immune Checkpoints in Cancer. Oncotarget.

[69] Latchman Y., Wood C.R., Chernova T., Chaudhary D., Borde M., Chernova I., Iwai Y., Long A.J., Brown J.A., Nunes R. (2001). PD-L2 Is a Second Ligand for PD-1 and Inhibits T Cell Activation. Nat. Immunol..

[70] Acúrcio R.C., Pozzi S., Carreira B., Pojo M., Gómez-Cebrián N., Casimiro S., Fernandes A., Barateiro A., Farricha V., Brito J. (2022). Therapeutic Targeting of PD-1/PD-L1 Blockade by Novel Small-Molecule Inhibitors Recruits Cytotoxic T Cells into Solid Tumor Microenvironment. J. Immunother. Cancer.

[71] Wang W., Zhang L., Sun Z. (2022). Eliciting Pyroptosis to Fuel Cancer Immunotherapy: Mechanisms and Strategies. Cancer Biol. Med..

[72] Vadhan-Raj S., Abonour R., Goldman J.W., Smith D.A., Slapak C.A., Ilaria R.L., Tiu R.V., Wang X., Callies S., Cox J. (2017). A First-in-Human Phase 1 Study of a Hepcidin Monoclonal Antibody, LY2787106, in Cancer-Associated Anemia. J. Hematol. Oncol..

[73] Poli M., Girelli D., Campostrini N., Maccarinelli F., Finazzi D., Luscieti S., Nai A., Arosio P. (2011). Heparin: A Potent Inhibitor of Hepcidin Expression in Vitro and in Vivo. Blood.

[74] Yu P.B., Hong C.C., Sachidanandan C., Babitt J.L., Deng D.Y., Hoyng S.A., Lin H.Y., Bloch K.D., Peterson R.T. (2008). Dorsomorphin Inhibits BMP Signals Required for Embryogenesis and Iron Metabolism. Nat. Chem. Biol..

[75] Katsarou A., Pantopoulos K. (2018). Hepcidin Therapeutics. Pharmaceuticals.

[76] Hawula Z.J., Wallace D.F., Subramaniam V.N., Rishi G. (2019). Therapeutic Advances in Regulating the Hepcidin/Ferroportin Axis. Pharmaceuticals.

[77] Laudisi F., Cherubini F., Monteleone G., Stolfi C. (2018). STAT3 Interactors as Potential Therapeutic Targets for Cancer Treatment. Int. J. Mol. Sci..

[78] Blanchette-Farra N., Kita D., Konstorum A., Tesfay L., Lemler D., Hegde P., Claffey K.P., Torti F.M., Torti S.V. (2018). Contribution of Three-Dimensional Architecture and Tumor-Associated Fibroblasts to Hepcidin Regulation in Breast Cancer. Oncogene.

[79] Formica V., Di Grazia A., Bonomo M.V., Frascatani R., Mancone R., Monteleone G. (2024). Circulating Hepcidin Levels Are an Independent Predictor of Survival in Microsatellite Stable Metastatic Colorectal Cancer Patient Candidates for Standard First-Line Treatment. Cancers.

